# The CFTR P67L variant reveals a key role for N-terminal lasso helices in channel folding, maturation, and pharmacologic rescue

**DOI:** 10.1016/j.jbc.2021.100598

**Published:** 2021-03-26

**Authors:** Carleen Mae Sabusap, Disha Joshi, Luba Simhaev, Kathryn E. Oliver, Hanoch Senderowitz, Marcel van Willigen, Ineke Braakman, Andras Rab, Eric J. Sorscher, Jeong S. Hong

**Affiliations:** 1 Department of Pathology, University of Alabama at Birmingham, Birmingham, Alabama, USA; 2 Department of Pediatrics, Emory University School of Medicine, Atlanta, Georgia, USA; 3 Department of Chemistry, Bar-Ilan University, Ramat-Gan, Israel; 4 Department of Cellular Protein Chemistry, Utrecht University, Utrecht, Netherlands

**Keywords:** cystic fibrosis transmembrane conductance regulator, lumacaftor, protein misfolding, protein trafficking, drug action, protein structure, CF, cystic fibrosis, CFTR, cystic fibrosis transmembrane conductance regulator, ER, endoplasmic reticulum, FBS, fetal bovine serum, FDA, Food and Drug Administration, FRT, Fischer rat thyroid, HEK293, human embryonic kidney 293, Lh1, lasso helix 1, Lh2, lasso helix 2, MD, molecular dynamics, NBD1, nucleotide-binding domain 1, NBD2, nucleotide-binding domain 2, TMD1, transmembrane domain 1, TMD2, transmembrane domain 2

## Abstract

Patients with cystic fibrosis (CF) harboring the P67L variant in the cystic fibrosis transmembrane conductance regulator (CFTR) often exhibit a typical CF phenotype, including severe respiratory compromise. This rare mutation (reported in <300 patients worldwide) responds robustly to CFTR correctors, such as lumacaftor and tezacaftor, with rescue in model systems that far exceed what can be achieved for the archetypical CFTR mutant F508del. However, the specific molecular consequences of the P67L mutation are poorly characterized. In this study, we conducted biochemical measurements following low-temperature growth and/or intragenic suppression, which suggest a mechanism underlying P67L that (1) shares key pathogenic features with F508del, including off-pathway (non-native) folding intermediates, (2) is linked to folding stability of nucleotide-binding domains 1 and 2, and (3) demonstrates pharmacologic rescue that requires domains in the carboxyl half of the protein. We also investigated the “lasso” helices 1 and 2, which occur immediately upstream of P67. Based on limited proteolysis, pulse chase, and molecular dynamics analysis of full-length CFTR and a series of deletion constructs, we argue that P67L and other maturational processing (class 2) defects impair the integrity of the lasso motif and confer misfolding of downstream domains. Thus, amino-terminal missense variants elicit a conformational change throughout CFTR that abrogates maturation while providing a robust substrate for pharmacologic repair.

The cystic fibrosis transmembrane conductance regulator (CFTR) is a chloride and bicarbonate ion channel and member of ABC-C gene family. Organ systems most severely affected by defects in CFTR comprise secretory epithelium, such as respiratory, gastrointestinal, and reproductive tissues. Predominant morbidity and mortality in the disease are attributable to pulmonary failure and associated defects in mucociliary clearance, chronic pulmonary infection, and airway remodeling (1, 2, 3, 4, 5).

Cystic fibrosis (CF) has been viewed as emblematic of emerging strategies for personalized medicine (4, 5)—a therapeutic modality in which clinical interventions are tailored to specific disease-causing mutations. Such initiatives have benefited enormously from recent advances in CF drug discovery and CFTR molecular characterization (5). In 2012, the potentiator ivacaftor was approved for patients harboring the CFTR gating mutation, G551D. Numerous additional gating defects and/or residual function variants have subsequently been Food and Drug Administration (FDA) registered for treatment with ivacaftor, extending the first CFTR-directed therapy. CFTR correctors, including lumacaftor, tezacaftor, and elexacaftor, are now FDA approved to ameliorate protein misfolding and premature degradation caused by deletion of phenylalanine at position 508 of the protein (termed F508del). Most CFTR trafficking defects (as well as WT CFTR) appear responsive to stabilization, enhanced surface localization, and increased function by these compounds (6), indicating potentially broad range activity for agents primarily developed to address a single genotype.

The present study focuses on P67L CFTR, a severe maturational processing defect that exhibits pronounced response to lumacaftor (manyfold higher than F508del) (6, 7, 8), although the underlying mechanism for this corrective feature is unknown. We used multiple protocols not previously applied to P67L and evaluated protein maturation, low-temperature rescue, and intragenic second-site suppressors (the latter to promote biogenesis by augmenting intradomain/interdomain conformation) (9, 10, 11). The proximity of P67 to a poorly understood “lasso” structure suggests a fundamental role for the motif (and particularly lasso helix 2 [Lh2]) during both CFTR assembly and pharmacologic repair. We tested this assertion by probing other lasso-localized mutations (known to cause CF) and studied their impact on protein trafficking, pharmacocorrection, nucleotide-binding domain 1 (NBD1) stabilization, and downstream effects as judged by pulse chase, limited proteolysis, and molecular dynamics (MD). Our findings provide new data relevant to folding and conformational effects of P67L on downstream CFTR domains, including structure/activity relationships for clinically important mutations in the CFTR amino terminus. MD simulations were used to evaluate mechanisms that underlie these results and point to a model in which the CFTR lasso region furnishes critical support for downstream domains of the protein, with drugs such as lumacaftor working at the amino terminus to stabilize full-length CFTR.

## Results

### Conservation of P67 and the lasso structure

**Figure 1:**
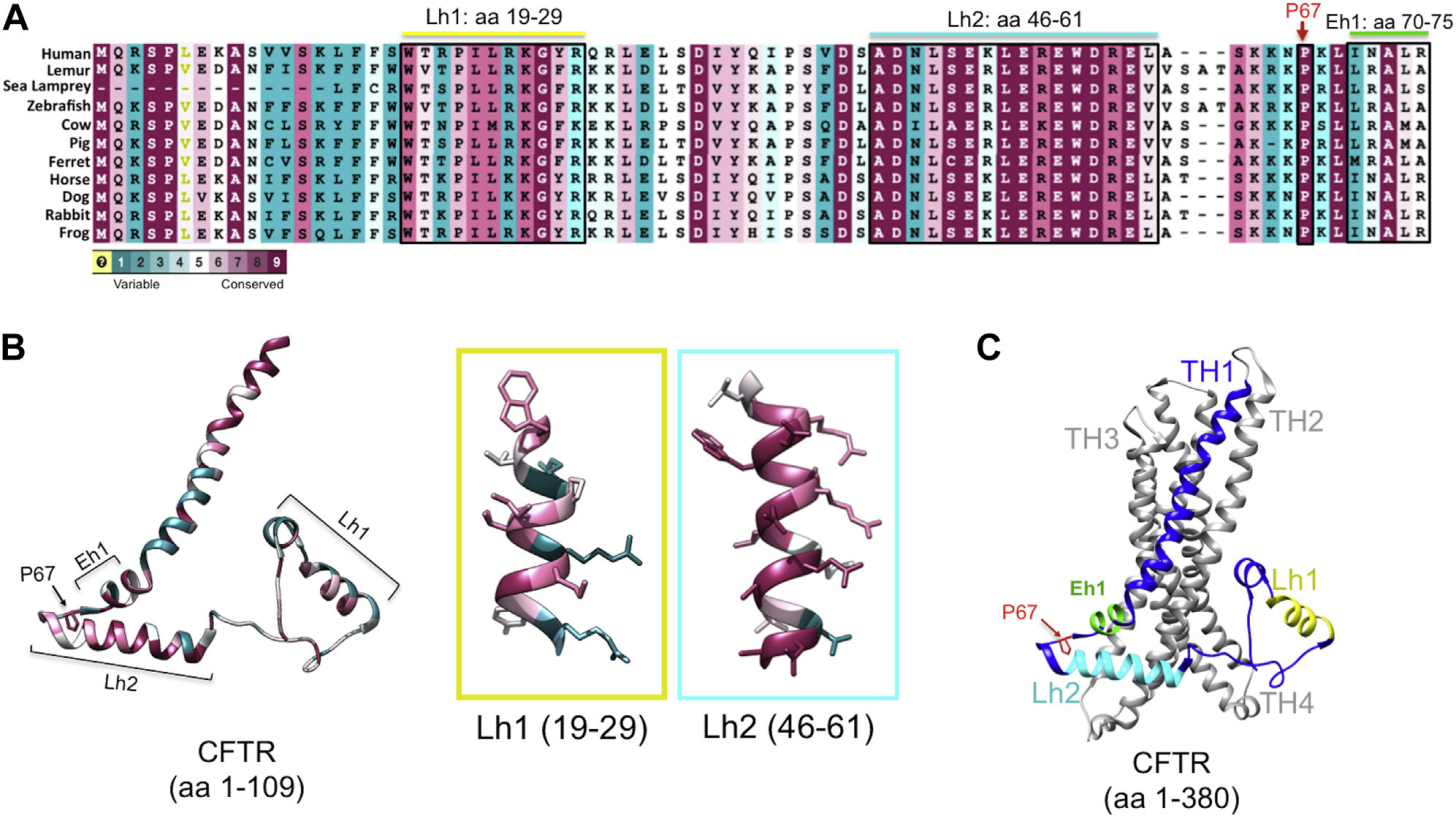
**Conservation of the lasso motif and P67 at the N terminus.***A*, alignment of CFTR amino terminus segment (aa 1–109) across multiple species, emblematic of a larger, ConSurf-based analysis ((72), Experimental procedures section). Color legend indicates highly variable (*light blue*) to average (*white*) to highly conserved (*magenta*) residues; *yellow* indicates insufficient data. *B*, residues conserved throughout species listed are highlighted in *magenta* (human CFTR; Protein Data Bank: 5UAK). *C*, the P67L variant is situated between lasso helix 2 (Lh2 shown in *light blue*) and elbow helix 1 (Eh1 shown in *green*) and in a region proximate to numerous transmembrane helices (THs) within CFTR membrane-spanning domains. CFTR, cystic fibrosis transmembrane conductance regulator.

The primary CFTR sequence was aligned among multiple species (Fig. 1*A*). P67, which occurs between Lh2 and elbow helix 1 based on the recently published cryo-EM structure of CFTR (12) (Fig. 1, *B* and *C*), is invariant among all species we evaluated. The two lasso helices and elbow helix are also highly conserved in all CFTR, from lamprey to human.

### Maturational profile of P67L CFTR, an Lh2 bordering mutation, in comparison to F508del

**Figure 2:**
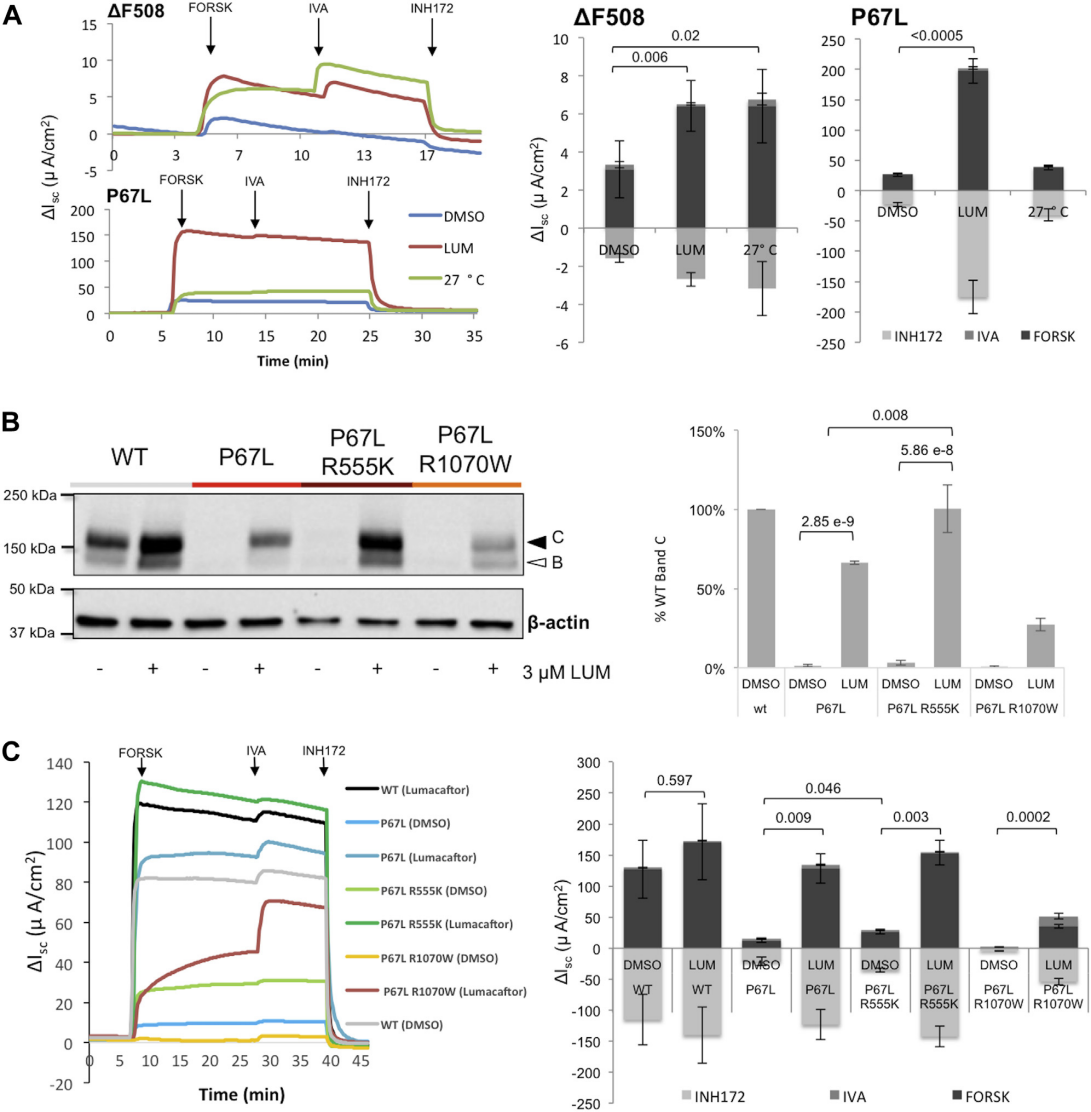
**P67L exhibits a molecular folding phenotype different from F508del.***A*, F508del lumacaftor functional correction in comparison to 27 °C (low temperature) rescue. Note change in *y*-axis in *upper versus lower left panels*. *Arrows* indicate forskolin (CFTR activator *via* cAMP/protein kinase A, 20 μM), ivacaftor (CFTR potentiator, 5 μM), and inh172 (CFTR inhibitor, 10 μM) (N = 6–9 filters/condition). Low-temperature incubation has been shown previously to confer diminished levels of WT CFTR expression (13). *B*, P67L band C (mature, post ER glycoform) in *cis* with R555K or R1070W second-site suppression following lumacaftor (N = 3 replicates/condition). Both second-site suppressors have been shown previously to rescue F508del processing (9, 10). Relative quantification (% wt band C) of the mature (mutant) glycoform shown on *left* was normalized to CFTR mRNA level in each sample (*right*). *C*, in Ussing chamber analysis, R555K was tested for effect on P67L activity. Short-circuit current tracings provided in the *left panel* are quantified on *right* (N = 3 replicates/condition). Acute additions were as previously mentioned except forskolin was tested at 10 μM. Lumacaftor was administered for 48 h at 3 μM prior to arrays shown. These studies were performed in the Fischer rat thyroid model. *p* Values indicate level of change as measured for band C CFTR or forskolin response (Student's *t* test). Error bars show mean ± SD (panel *A* and *B*) or mean ± SEM (panel *C*). CFTR, cystic fibrosis transmembrane conductance regulator.

The F508del mutation is associated with reduced CFTR expression at the cell surface because of protein misfolding and endoplasmic reticulum (ER)–associated degradation as well as reduced stability of the final folded state (10). F508del exhibits improved CFTR maturation (increased band C glycoform) in response to incubation at 27 °C (13). Lumacaftor confers similar levels of F508del correction to low temperature as judged by Ussing chamber analysis (twofold increase in chloride transport) in an epithelial model widely utilized for studies of CFTR biogenesis and drug discovery (Fischer rat thyroid [FRT] cell model), typically used for CF drug screening applications, mechanistic studies, and expansion of FDA drug approvals in the United States (7, 8, 14, 15, 16, 17) (Fig. 2*A*). In contrast, although F508del functional rescue by low temperature is roughly comparable to what can be achieved with lumacaftor, P67L chloride transport after 27 °C incubation is only ∼20% of lumacaftor correction. We next introduced second-site suppressors known to rescue F508del and investigated the corrective effect on P67L. Incorporation of the R1070W second-site suppressor (which rescues F508del by restoring an intracellular loop 4/NBD1 interface (10, 11)) does not improve P67L CFTR maturation (Fig. 2*B*). Conversely, R555K (which promotes NBD1 stabilization (9, 10, 11, 18)) partially rescues P67L CFTR function (Fig. 2*C*), with modest enhancement of band C following treatment with lumacaftor. R555K has been shown previously to improve solubility of isolated CFTR NBD1, enhance expression/yield of NBD1, rescue F508del CFTR through stabilizing effects on the first NBD, and evolutionarily couple with other key residues in CFTR NBD1 (10, 19). Results shown in Figure 2*B* (together with data described later) collectively suggest that P67L may allosterically disrupt structural integrity of downstream CFTR domains, an effect that can be rescued by an NBD1 suppressor mutation. Findings in Figure 2 also demonstrate that a mutation resistant to 27 °C rescue nevertheless can be strongly corrected by lumacaftor (VX-809) (to levels much higher than F508del CFTR) and provide new evidence that low-temperature rescue and VX-809 pharmacocorrection improve F508del CFTR processing through distinct mechanisms. Moreover, a clinically important mutation near the lasso helical domain of CFTR confers defective maturation that can be overcome by stabilizing NBD1 through R555K.

### N-terminal CFTR mutations exhibit distinct lumacaftor response

**Figure 3:**
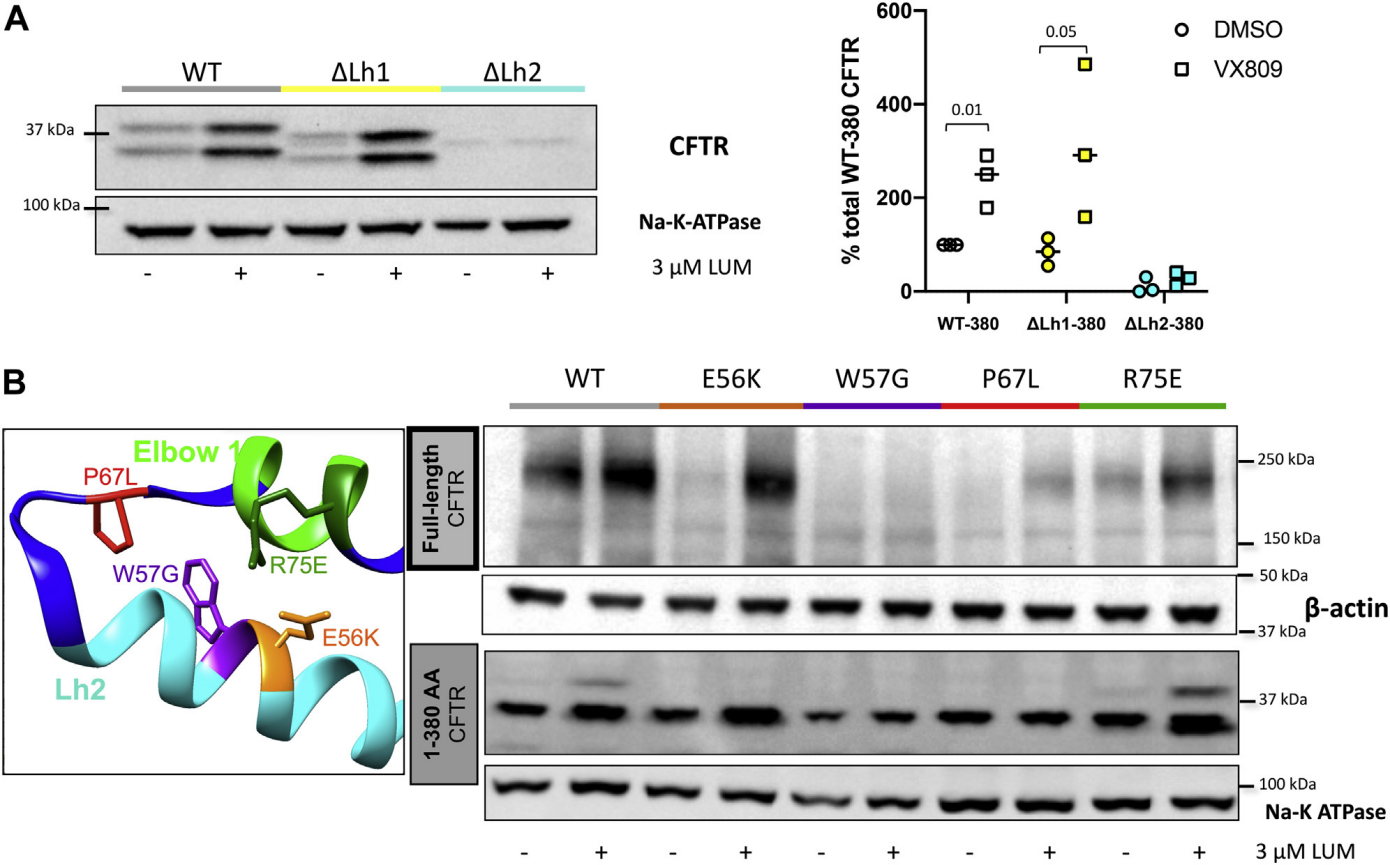
**Lasso helix 2 is necessary for CFTR maturation.***A*, construct encoding the first 380 residues of CFTR was used to probe modulator response of TMD1 (6) following deletion of Lh1 (Lh1del, aa 19–29) or loss of Lh2 (aa 46–61) (n = 3) during transient expression in human embryonic kidney 293 cells. *p* Values by Student's *t* test. *B*, response to lumacaftor (3 μM, 24 h incubation) of CFTR-380 (TMD1) constructs in human embryonic kidney 293 cells was measured following introduction of specific N-terminal point mutations. (P67L, E56K [within Lh2], R75E [a mutation near Lh2 at the tail of elbow helix 1], or W57G [a point mutation within Lh2]). Key aspects of this experiment have been repeated 3 to 5 times with similar results. Detection of CFTR was with MM13-4 antibody. CFTR, cystic fibrosis transmembrane conductance regulator; TMD, transmembrane domain.

In order to further characterize P67L as part of the N-terminal lasso structure, truncated CFTR encoding the first 380 amino acids was generated to include a series of mutations localized in the region of Lh1 and Lh2 (Fig. 3). As previously reported, WT CFTR-380 (encoding CFTR transmembrane domain 1 [TMD1]) exhibits detectable levels of protein that can be enhanced further with lumacaftor (6). CFTR-380 without Lh1 exhibits a similar molecular phenotype to WT CFTR-380. Omission of Lh2 from this construct, however, is associated with barely detectable protein and complete loss of lumacaftor-mediated protein stabilization (Fig. 3*A*). A doublet is observed for the CFTR-380 construct, potentially because of changes in globular protein structure during polyacrylamide gel electrophoresis. Very distinct effects of Lh-deletion constructs indicate that CFTR destabilization by omission of Lh2 is more severe than Lh1.

The clinically important CFTR variants E56K, W57G, P67L, as well as a nearby (nondisease associated) mutation, R75E, were introduced into full-length or truncated CFTR (Fig. 3*B*) (https://cftr2.org/) (20, 21, 22). E56K exhibits robust lumacaftor response, whereas the immediately adjacent variant W57G (a mutation associated with severe CF disease) was refractory to pharmacocorrection (6, 22). The finding that a clinically important point mutation in CFTR (W57G) abrogates both protein trafficking and lumacaftor rescue further indicates the critical importance of Lh2 during CFTR maturational processing. The nearby elbow helix 1 mutation R75E demonstrates a similar profile to E56K. Levels of P67L, which occurs between Lh2 and elbow helix 1, are increased by lumacaftor in full-length CFTR. In the context of TMD1 alone, however, no increase in steady-state levels of P67L CFTR-380 was observed. Mutations of the CFTR N terminus therefore influence not only CFTR interdomain interaction but also structural requirements for lumacaftor activity and/or binding. Mutations in the lasso helical region, including those at positions less than 10 residues apart, can elicit nearly identical effects on overall CFTR maturational efficiency, yet exhibit pronounced differences with regard to pharmacocorrection (Fig. 3*B*).

### W57G is resistant to pharmacologic correction

A majority of class II (misfolding) CFTR mutations demonstrate at least modest levels of lumacaftor responsiveness (6, 17). To further characterize the lumacaftor-resistant variant W57G localized within Lh2, R555K was introduced in *cis*. Unlike P67L (which exhibits partial rescue following either lumacaftor treatment or R555K suppression), W57G—a mutation found in a small number of patients worldwide (CFTR2 database [https://cftr2.org/])—exhibits negligible effect on either protein maturation or CFTR chloride transport following these maneuvers (Fig. S1). P67L maintains a strong response to lumacaftor (6, 7, 23), and the nearby R75E variant (eight residues downstream) is also robustly rescued by the drug. Conversely, no response whatsoever to lumacaftor or R555K was observed for the W57G variant (10 amino acids upstream of P67) (Fig. 3*B* and Fig. S1).

### Lumacaftor-corrected P67L “half CFTR” exits the ER in the presence of downstream CFTR domains

**Figure 4:**
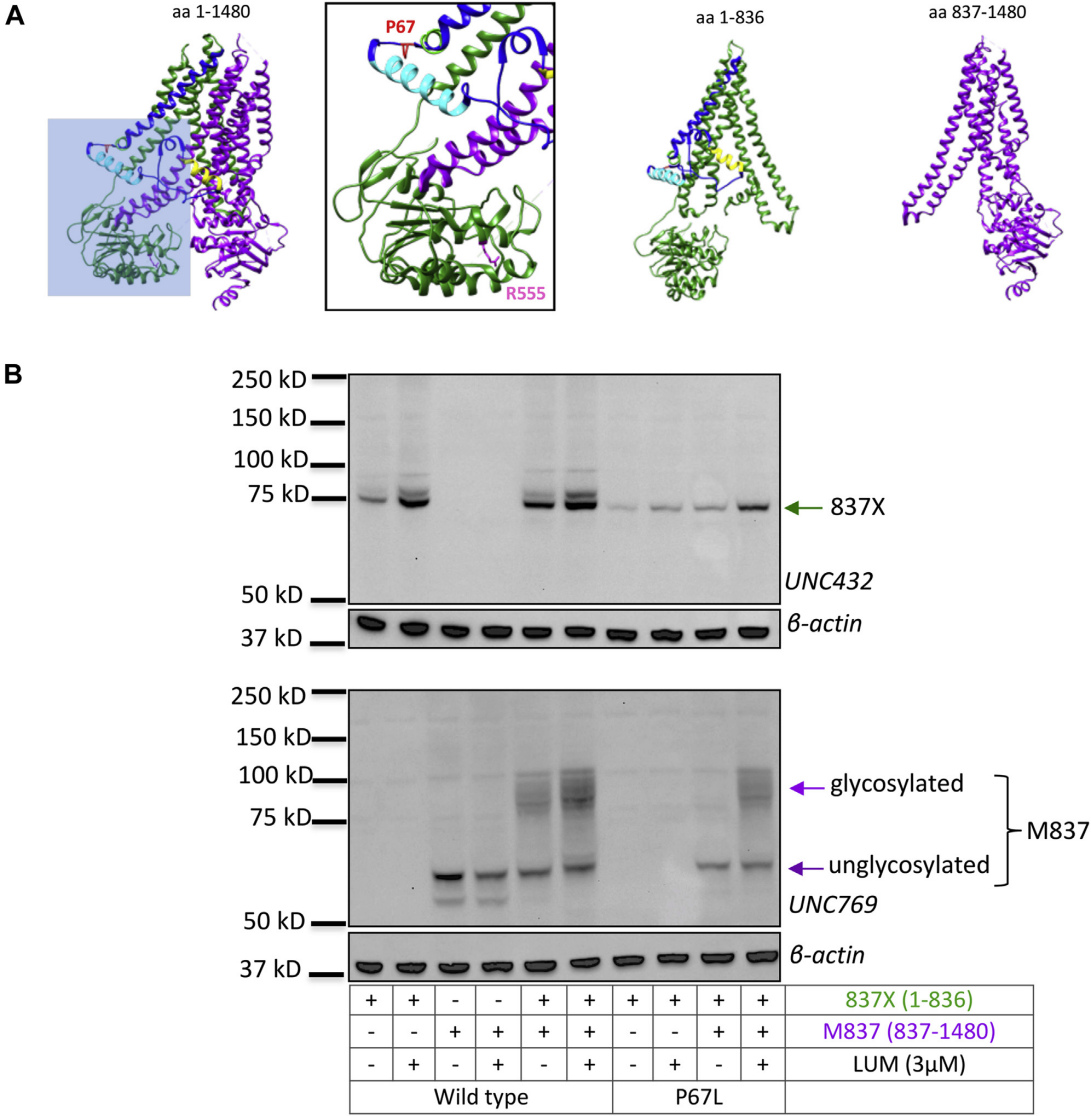
**Lumacaftor correction of P67L in the presence of the second half of CFTR.***A*, ribbon diagrams of human CFTR showing location of R555 and CFTR half molecules. *Blue inset* is expanded on *right*. In panel (*B*), protein stability is tested following lumacaftor treatment (3 μM, 24 h incubation) of the WT-837X construct alone or in the presence of M837 CFTR. P67L–837X was also evaluated following lumacaftor with M837 CFTR coexpressed. Studies were conducted in human embryonic kidney 293 cells, and CFTR was detected with UNC432 and UNC769 antibodies. This experiment has been repeated three times with similar results. CFTR, cystic fibrosis transmembrane conductance regulator.

As an additional test of P67L CFTR response to lumacaftor, a “half molecule” construct truncated near the R-domain C terminus (CFTR 837X; residues 1–836) was coexpressed with the downstream half of the molecule (M837; residues 837–1480) (Fig. 4) (24). WT-837X CFTR constructs studied in this manner (similar to WT CFTR-380) retain lumacaftor sensitivity, whereas the second half of CFTR, by itself, is unresponsive (Fig. 4*B*). In semblance to P67L CFTR-380, P67L–837X alone is stabilized following drug treatment. Increased steady-state levels of both P67L–837X and M837 CFTR are observed when both halves of the protein are present, including enhancement of the mature M837X glycoform (Fig. 4*B*). These results indicate that correct N-terminal conformation enhances CFTR processing and lumacaftor response in a fashion that requires proper domain interactions between the two half molecules.

### Limited proteolysis demonstrates downstream CFTR destabilization by P67L

**Figure 5:**
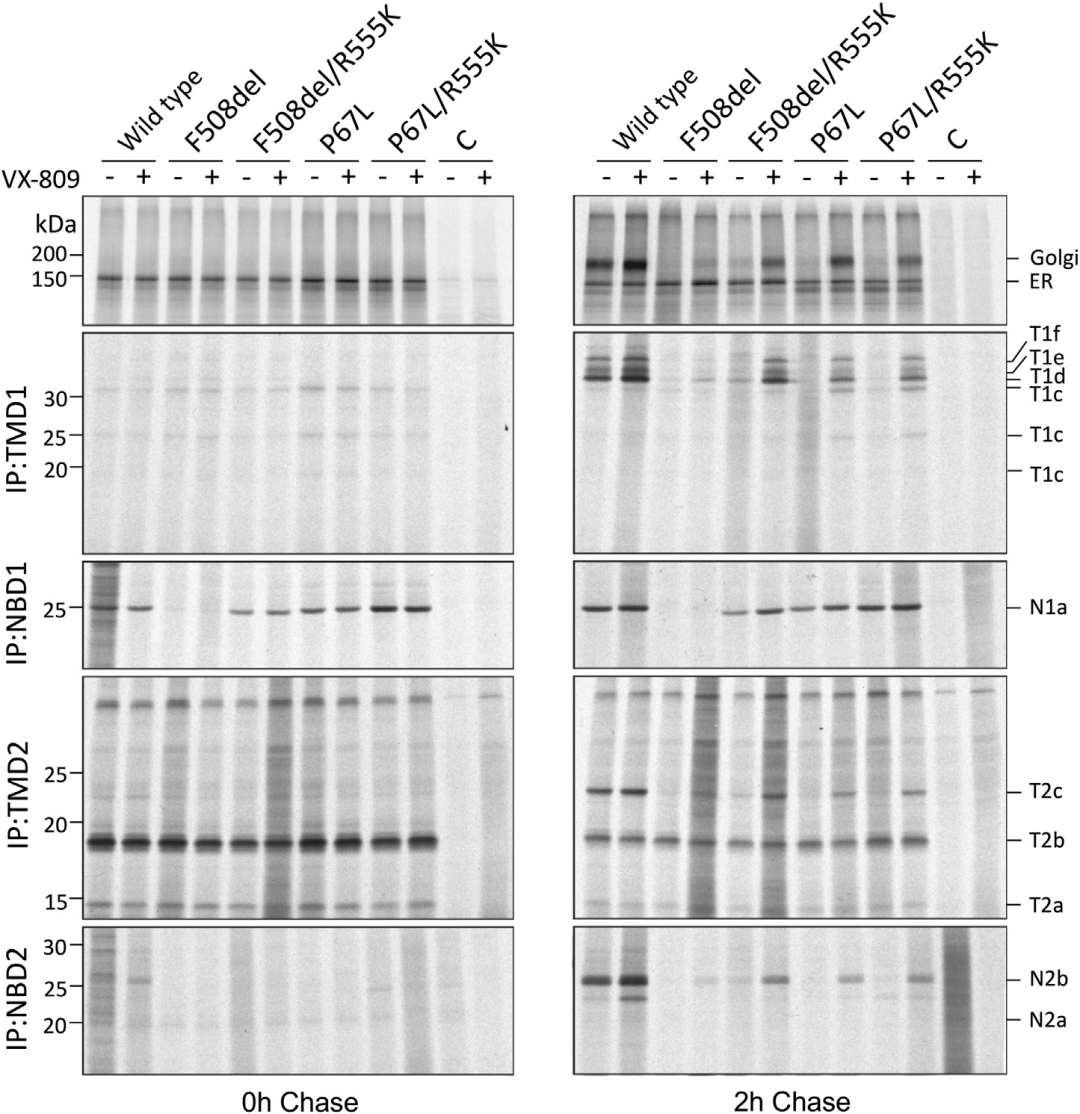
**P67L affects late domain folding.** Human embryonic kidney 293 cells were radiolabeled for 10 min and chased for 0 (*left panels*) or 2 h (*right panels*) in the presence or the absence of 3 μM VX-809 (lumacaftor). Detergent cell lysates were treated (*bottom four panels*) or not (*top panels*) with 25 μg/ml proteinase K. CFTR and fragments were immunoprecipitated with domain-specific antibodies: TMD1C (TMD1), Mr. Pink (which detects NBD1 fragments and nonproteolyzed CFTR NBD1) (30, 31, 32), TMD2C (TMD2), and 596 (NBD2) to probe conformation of each domain. Fragments are identified by domain according to nomenclature reported previously (31, 32). C, control transfection with empty plasmid; CFTR, cystic fibrosis transmembrane conductance regulator; NBD, nucleotide-binding domain; TMD, transmembrane domain.

To further investigate effects of P67L on CFTR folding, we conducted a protease-sensitivity assay using radiolabeled human embryonic kidney 293 (HEK293) cells expressing CFTR variants. In particular, we compared the P67L variant to F508del and assessed impact of R555K intragenic suppression and lumacaftor rescue. Immediately following a pulse label of 10 min, CFTR is predominantly localized to the ER or early Golgi (Fig. 5, *left panel*) (25, 26, 27, 28, 29), whereas a large pool of WT CFTR matures in the Golgi following a chase of 2 h (*right panel*). P67L and F508del CFTR do not leave the ER but misfold and are degraded (30, 31, 32). Both mutants were rescued partially by the intragenic suppressor R555K, which is strongly enhanced by lumacaftor. The moderate increase in newly synthesized P67L by R555K compared with the strong increase in steady-state levels suggests that R555K predominantly increased P67L CFTR folding and stability at the cell surface.

To probe conformation of all variants, we determined protease sensitivity of each CFTR domain. In general, the better folded a domain, the more protease-cleavage sites are shielded and the more protease resistant the domain will appear (30, 31, 32, 33, 34, 35). WT CFTR is digested reproducibly into protease-resistant fragments (nomenclature for the proteolytic fragments of TMD1, NBD1, transmembrane domain 2 [TMD2], and nucleotide-binding domain 2 [NBD2] is described) (31, 32). Except for NBD1, the fragments increase in size between the 0 and 2 h time points because of maturation of CFTR, leading to increasing compactness and protease resistance. Part of the N2b fragment at times becomes cleaved into a slightly smaller fragment, running below N2b (immunoprecipitation: NBD2). F508del not only lacks the NBD1 fragment because of misfolding of NBD1 but also lacks the later larger fragments of the other three domains (31) (Fig. 5).

F508del and P67L CFTR have similar protease sensitivities for TMD1, TMD2, and NBD2 at both time points (31, 32). However, F508del NBD1 is misfolded and hence completely protease sensitive, whereas P67L NBD1 resembled the WT CFTR proteolytic profile (Fig. 5; immunoprecipitation: NBD1). Following introduction of R555K on the F508del or P67L background, NBD1 folding was improved. In contrast, R555K in *cis* did increase neither protease resistance of TMD1 and TMD2 nor NBD2 under conditions shown here (2-h chase period). TMD1 yielded a barely detectable quantity of WT-similar fragments T1d-f (Fig. 5), suggesting that rescue of NBD1 by R555K partially corrected F508del but not P67L TMD1. WT CFTR yields a protease-resistant NBD2 fragment (N2b) after 2 h of chase, which in F508del and P67L CFTR is absent.

In the presence of VX-809, TMD1, TMD2, and NBD2 strongly increased protease resistance, albeit not to WT levels. VX-809 uncovered the effects of the R555K mutation in *cis*, showing a similar but stronger effect on F508del than on P67L. Our results indicate destabilization of multiple CFTR domains conferred by either the F508del mutation or the P67L mutation. The findings suggest that R555K corrects folding of F508del NBD1 and subsequently improves conformation of NBD2 (and perhaps also TMD1 and TMD2), especially in the presence of lumacaftor. The effect of R555K on P67L involves an improvement of NBD1 stability leading to enhancement of NBD2 folding.

### Evaluation of the lasso region by MD

**Figure 6:**
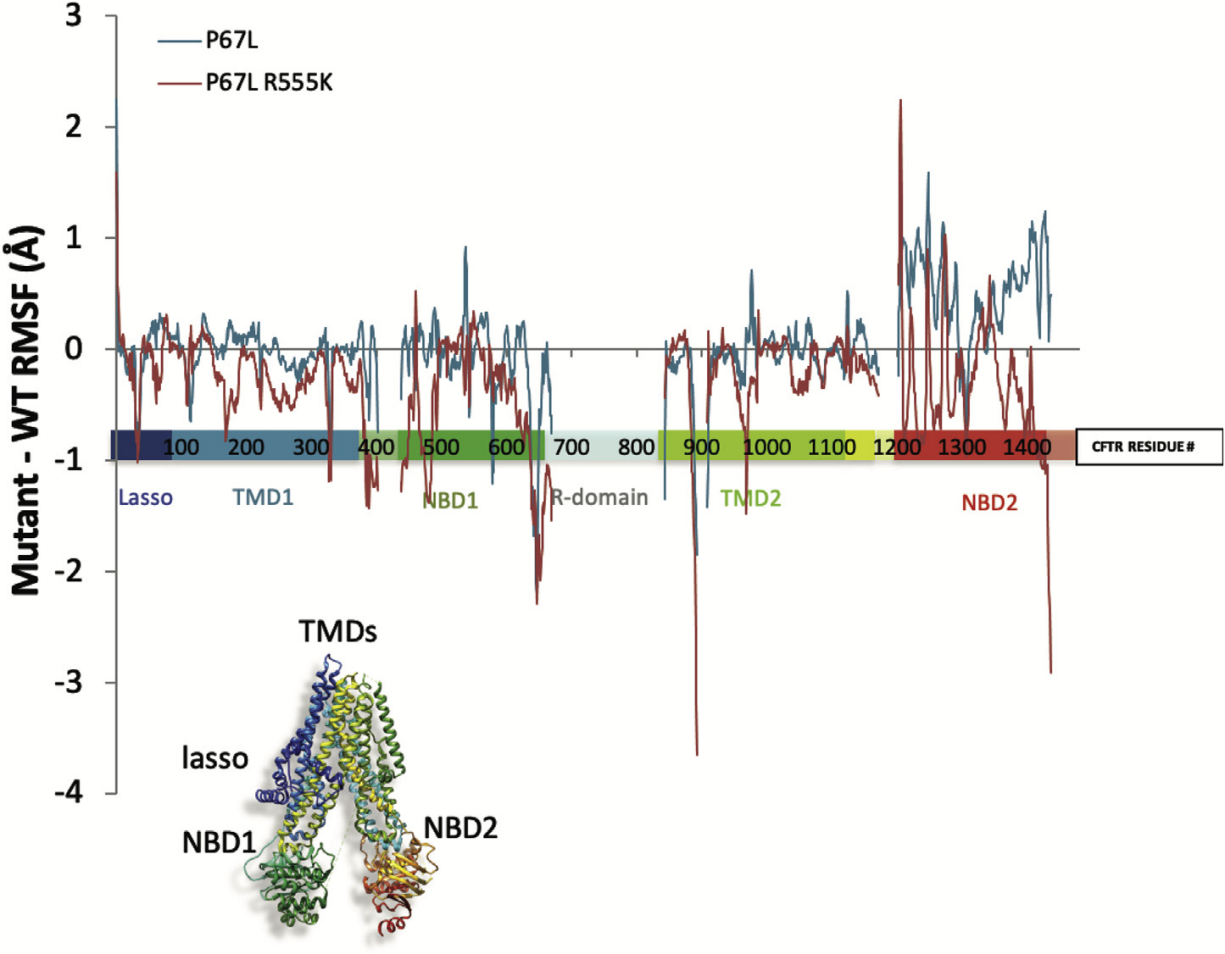
**R555K rescues P67L NBD2 destabilization in the inward model.** Analysis of averaged (over three 100 ns simulations) root-mean-square fluctuation difference profiles comparing fluctuations of WT *versus* P67L-CFTR (P67L—WT in *blue*) and of WT *versus* P67L/R555K-CFTR (P67L/R555K—WT in *red*) for inward-facing CFTR. Positive and negative values correspond to regions where the mutants are fluctuating more or less than the WT, respectively. P67L destabilizes NBD2 (∼1200–1400 region), whereas the R555K mutation appears to counteract this effect. Statistically significant differences were noted in the NBD2 region for WT *versus* P67L CFTR, as well as for P67L *versus* P67L/R555K CFTR (*p* = 0.05, *t* test with two samples assuming unequal variances). CFTR, cystic fibrosis transmembrane conductance regulator; NBD, nucleotide-binding domain.

**Figure 7:**
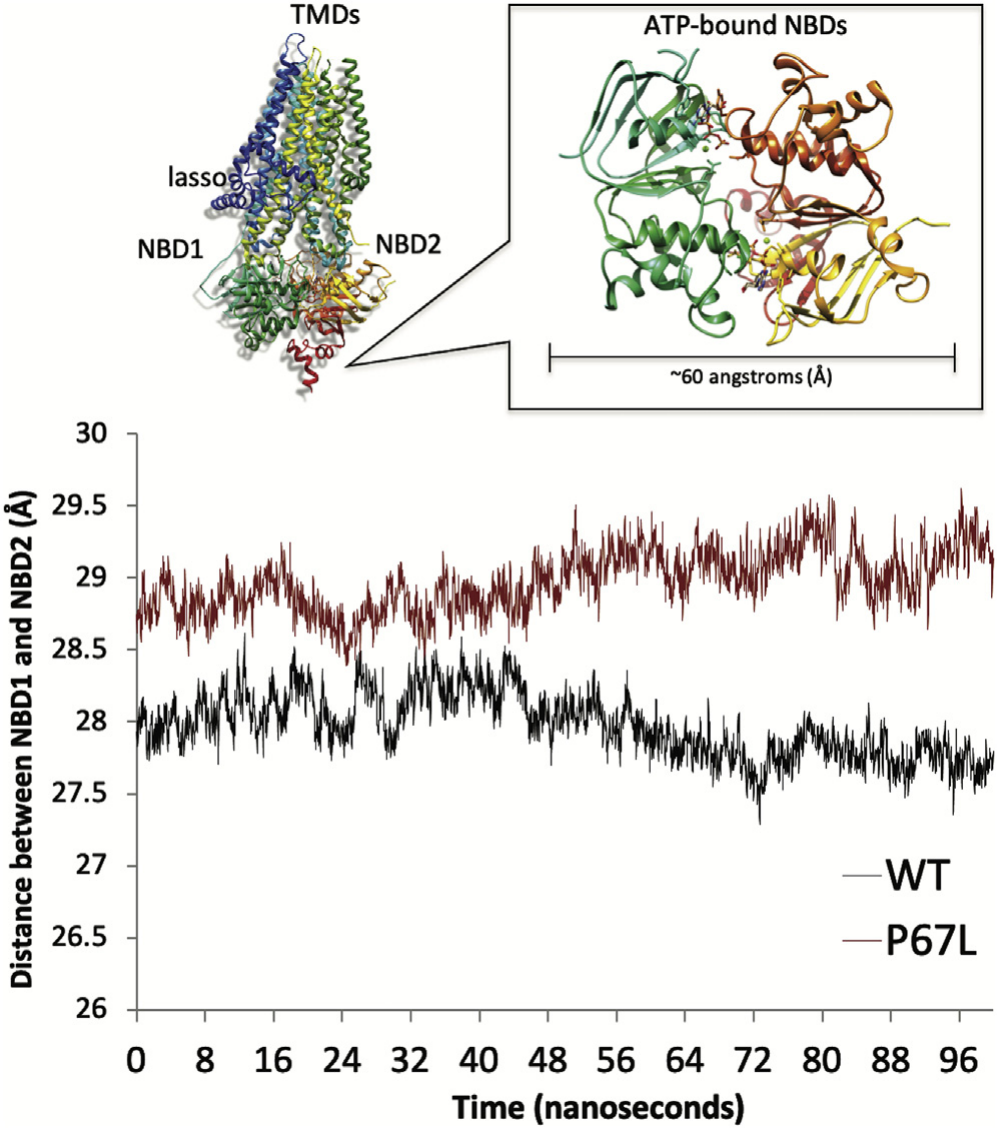
**Molecular dynamics simulations of P67L-CFTR exhibit increased molecular separation between NBDs.** Averaged (over three 100 ns simulations) distances between NBD1–NBD2 centers of mass derived from molecular dynamics simulations of models of WT (*black*) and P67L-CFTR (*red*) in the phosphorylated, ATP-bound, outward-facing conformation. Larger distances are clearly apparent for the P67L mutant and were found to be statistically significant at the *p* = 0.05 level (Student's *t* test). CFTR, cystic fibrosis transmembrane conductance regulator; NBD, nucleotide-binding domain.

**Figure 8:**
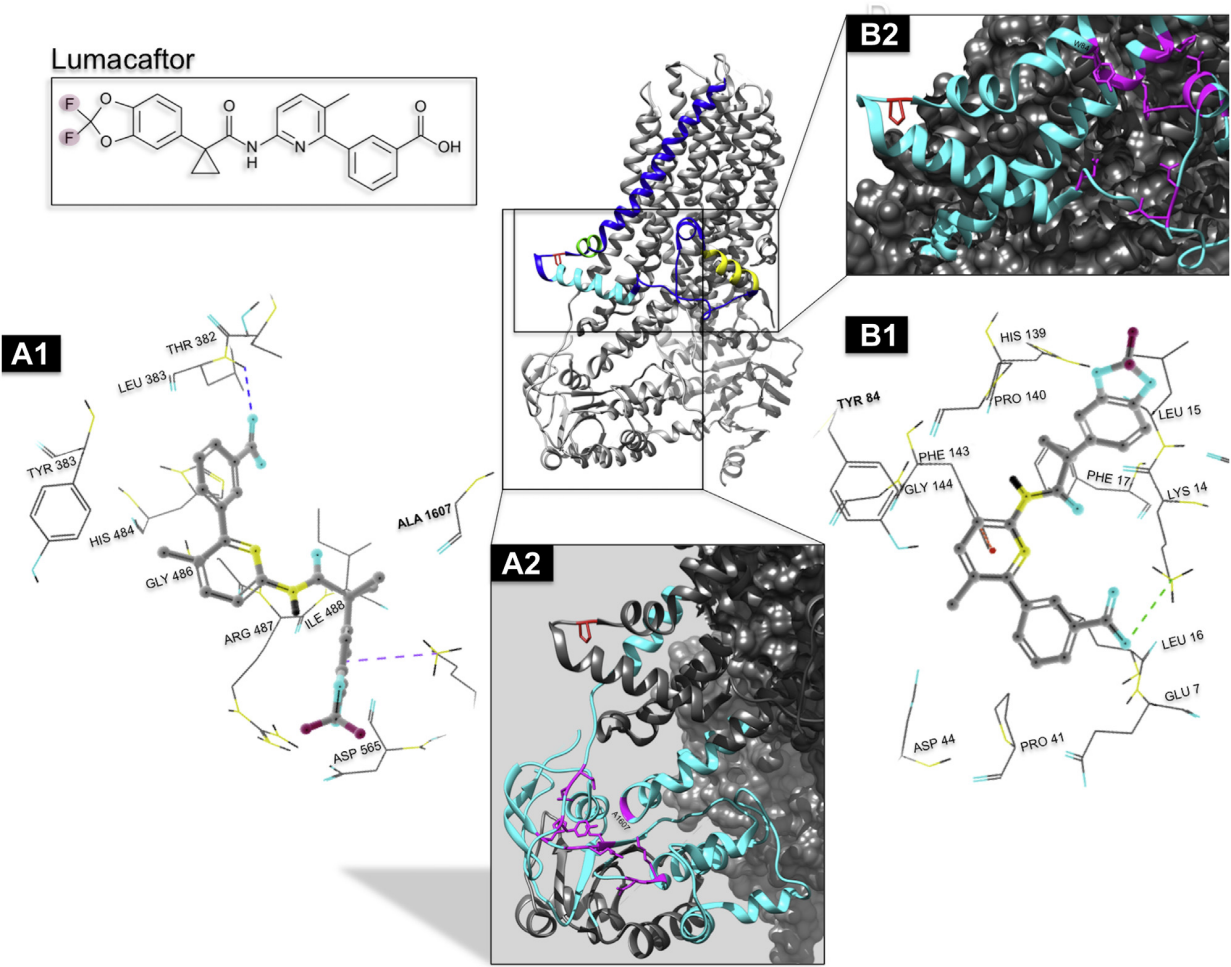
**Potential binding sites for lumacaftor flank Lasso helix 2.** A model of the inward-facing cystic fibrosis transmembrane conductance regulator and the lumacaftor chemical structure is depicted. *Insets* focus on predicted binding sites for lumacaftor; potential interacting residues are highlighted in *magenta*. A1 and A2 suggest lumacaftor localization in proximity of the ICL4–NBD1 interface, adjacent to the end of transmembrane helix 6 (TMH6) (∼residue Y380). B1 and B2 portray an alternative binding site within the lasso region and involving the n terminus upstream of amino acid residue 80, transmembrane helices 1 and 2.

Previous studies have simulated various aspects of CFTR dynamics based on the WT protein structure or on models of CFTR derived from homology to other ABC transporters (*e.g.*, SAV1866, P-gp, McjD, ABC-B10, TM-287-288) (36, 37, 38, 39, 40, 41, 42). In the present work, we utilized cryo-EM–derived structures of zebrafish WT-CFTR (12, 43) in two different conformations to build corresponding homology models of various human CFTR constructs (WT, P67L, and P67L/R555K-CFTR) and subjected them to MD simulations (Fig. S2). Averaged root-mean-square deviation of atomic position plots for the distinct constructs demonstrates simulation convergence. We examined the root-mean-square fluctuation profiles of the resulting trajectories as a mean to evaluate protein stability. We have previously shown a favorable correlation between fluctuation profile and thermal stability for a series of NBD1 constructs (44), whereas correlation between fluctuation and stability for additional proteins has been observed by others (45). In the present analysis, P67L appears to markedly destabilize NBD2 (as demonstrated by increased regional fluctuation relative to WT) (Fig. 6; see also Figs. S3 and S4). Simulations including the second-site suppressor R555K in *cis* with P67L exhibit a striking and complete reversal of predicted changes in protein stability attributable to P67L. Notably, analysis of the P67L NBD1–NBD2 center of mass distance based on simulating a phosphorylated ATP-bound model indicates enlarged separation between the two domains consequent to P67L mutation, which in turn suggests lower binding affinity between the NBDs (Fig. 7). Finally, the P67L trajectories were analyzed using the MDpocket algorithm (46) in search of putative lumacaftor-binding sites, and a distinct site immediately adjacent to Lh2 was observed (Fig. 8).

## Discussion

### Earlier analysis concerning the CFTR N terminus

The CFTR amino terminus has been shown previously to participate during regulation of ATP-dependent gating and endocytosis, with mutations in this region leading to defects in channel opening (47, 48). D58N, a CF-associated mutation in Lh2, for example, was reported to impair macroscopic chloride transport as judged by studies in *Xenopus* oocytes (49). Point mutations of acidic residues adjacent to D58 may also disrupt channel gating. These functional effects appear to be determined in part by a negatively charged surface of the amino terminus (50), perhaps interacting with the R domain. The same structure has been suggested to bind membrane-anchoring proteins such as syntaxin 1A (49, 51). Previous studies therefore indicate importance of the CFTR amino terminus during ion channel gating (see also later).

### The N terminus contains a lasso motif adjacent to P67

CFTR structural data (52) provide a means to evaluate both the basic P67L molecular defect as well as lumacaftor mechanism of action. Prolines often confer a structural turn within alpha helices (53), and disruption of this turn can result in the absence of a key peptide angle and significant misfolding (54). Four-residue turn motifs in the CFTR amino terminus may also serve as mediators of protein misfolding (*e.g.*, amino acids constituting classic beta turns both upstream (S63, K64, K65, and N66) and downstream (K68, L69, I70, N71, and C76) of P67). While homology models have successfully predicted the organization of CFTR pore-forming elements and ATP-binding domains (using reference proteins such as p-glycoprotein and SAV1866) (55, 56, 57, 58, 59), identification of the N-terminal lasso motif in proximity to P67L was only revealed by cryo-EM of CFTR, itself (52). The lasso is absent among other ABC-C proteins and CFTR analogs (much like the CFTR-restricted R domain (60, 61)). Moreover, as suggested by its name, the lasso appears to physically constrain a number of transmembrane helices and has been suggested to partially insert within the plasma membrane (Fig. 1*C*). The motif is therefore distinct from other lasso-type regions, which arise when termini of a peptide pierce surfaces of minimal area covered by a covalent loop (62). The findings presented here indicate pronounced dependence of CFTR biogenesis on an intact Lh2 and are among the first to elucidate a specific role for this structure during CFTR maturational arrest.

### The amino terminus is crucial for lumacaftor activity and overall CFTR folding

Lumacaftor-dependent peptide stabilization is preserved in constructs containing the first 380 amino acids of CFTR (6). In order to elucidate significance of this region (and specifically, the constituent lasso), we established truncated CFTR-380 lacking Lh1 and Lh2 (Lh1del and Lh2del, respectively). Lh1del exhibited baseline levels of CFTR comparable to WT and strongly responsive to lumacaftor. In contrast, Lh2del CFTR was undetectable both at baseline and following modulator treatment. Using the CFTR2 patient genotype database (https://cftr2.org/, which is intended to rigorously define CFTR mutations that cause disease), we identified clinically relevant mutations located within 5 Å of Lh2, including two variants immediately adjacent to each other. E56K is a partial-function mutation clinically approved for ivacaftor administration ((8), https://www.cff.org/News/News-Archive/2017/FDA-Approves-Ivacaftor-for-23-Additional-CFTR-Mutations/ (Accessed: December 21, 2020)) and often associated with pancreatic sufficiency. Patients harboring W57G, on the other hand, are predominantly pancreatic insufficient. Despite structural proximity, E56K confers a class II (protein maturation) defect with WT levels of CFTR following lumacaftor, whereas W57G is completely resistant to the compound. R75E, a mutation opposite W57G on the neighboring elbow helix 1, exhibits higher levels of residual (mature) CFTR and a measure of lumacaftor susceptibility. P67L occurs within a strand of amino acids connecting Lh2 and elbow helix 1, allowing the elbow to align with TMDs in a manner antiparallel to Lh2. Our findings establish marked sensitivity of CFTR folding and maturational processing to specific point mutations in the amino terminus and Lh2 in particular.

### TMD1 stabilization by lumacaftor is dependent on intact Lh2

When we introduced N-terminal mutations into truncated CFTR-380 and tested impact on TMD1 in the setting of lumacaftor binding, several constructs exhibited a response resembling the effect on full-length CFTR. In this context, CFTR-380 findings should be interpreted in light of data that support lumacaftor binding to the amino portion of CFTR at an ICL4–NBD1 interface (13, 63, 64), particularly since neither ICL4 nor NBD1 is present in the TMD1 construct. Recent simulations place the lumacaftor-binding site near Lh2 in the region of TMD/NBD interaction, rather than directly at ICL4–NBD1, itself (65). Our findings support a model in which both P67L CFTR misfolding and lumacaftor repair impact CFTR conformation, with activity of the small molecule requiring key determinants of TMD1, including the Lh2.

### Mutations in the N terminus impact downstream protein domains

The finding that full-length CFTR encoding P67L is strongly corrected by lumacaftor, but that CFTR-380del encoding P67L exhibits minimal stabilization (compared with other 380del constructs), suggests that elements downstream of CFTR position 380 are essential during lumacaftor rescue of P67L CFTR. Pathogenic effects resulting from F508del (9, 10, 11, 66) include alterations of NBD stability and diminished binding between NBD1 and ICL4. Second-site suppressors within NBD1 (R555K) and at the interdomain interface (R1070W) counteract these abnormalities in a specific manner (9, 10, 67, 68, 69). Transiently expressed constructs encoding R555K (but not R1070W) in *cis* with P67L exhibited rescue of the mature protein, indicating P67L maturation can be enhanced through stabilizing effects on NBD1.

Limited proteolysis provides further evidence for multidomain abnormalities attributable to P67L. R555K appears to correct NBD1 folding as judged by proteolytic pattern and thereby enhances steady-state levels of a prominent NBD1 fragment for both P67L and F508del. The R555K second-site suppressor also improves NBD folding of both P67L and F508del (Fig. 5). In apparent contrast to steady-state Western blot data (Fig. 2), pulse chase of P67L CFTR does not show a marked increase in ER and Golgi forms by R555K. This implies that the increased quantity of P67L–R555K in Western blot steady state (Fig. 2), while starting with only slightly improved biosynthesis in the ER, stems from the resulting increased stability at the cell surface.

Our biochemical results are consistent with MD simulations demonstrating a decrease in stability of NBD2 mediated by P67L and improvement of this by R555K (Fig. 6). Domains significantly downstream of P67 therefore appear disrupted by P67L unfolding and lead to CFTR misprocessing. Our data also support the notion that lumacaftor works through stabilizing effects on multiple domains within CFTR to bring about pharmacologic correction. Notably, structural analysis identified a potential binding site for lumacaftor in the vicinity of sequence positions 370 to 380 and Lh2 (Fig. 8). Such findings are consistent with a model in which lumacaftor interacts with residues of TMD1 adjacent to the lasso, in agreement with earlier studies that suggest binding to this general region (6, 63, 65, 70, 71).

### Rescue profiles of P67L differ from F508del

In comparison to P67L, F508del responds less robustly to lumacaftor and improves after R555K (but not R1070W) second-site suppression. One interpretation of this finding is that P67L primarily requires rescue in TMD1, the domain that is mutated, and which lumacaftor targets. F508del CFTR is rescued less by lumacaftor (and retains the NBD1 defect) but responds more completely to second-site suppression (additive to the lumacaftor effect) because these steps rescue distinct defects in F508del. From that perspective, lumacaftor appears to rescue P67L CFTR by a mechanism different from either second-site suppression (R555K and R1070W) or low-temperature repair (Fig. 2). Another difference between P67L and F508del is that F508del appears to be rescued more strongly at the level of folding in the ER (pulse chase), whereas P67L required correction to leave the ER but was improved in a fashion that suggests increased cell-surface stability. The observation that lumacaftor binds TMD1 and can stabilize CFTRs with mutations in the amino terminus, NBD1, as well as more distal CFTR domains provides evidence that the effect of the compound on TMD1 also applies to many other class II variants.

### Summary

Our findings provide new evidence that the CFTR N terminus imposes a global influence on protein folding, biogenesis, and gating. We show importance of the lasso domains during maturational processing. While truncated CFTR-380 constructs missing Lh1 exhibit WT levels of CFTR peptide, deletion of Lh2 disrupts both protein expression and lumacaftor activity. Furthermore, a single mutation within Lh2 (W57G) completely abolishes CFTR trafficking to the cell surface and lumacaftor rescue. Because large numbers of mutations in the CFTR2 database (see previous)—as well as WT CFTR—respond to lumacaftor, one can argue that residues such as W57 and/or Lh2 could contribute directly to binding of lumacaftor within TMD1. On the other hand, loss of lasso components may elicit local and/or allosteric conformational changes that disrupt the lumacaftor-binding pocket or otherwise prevent lumacaftor-induced conformational rescue.

**Figure 9:**
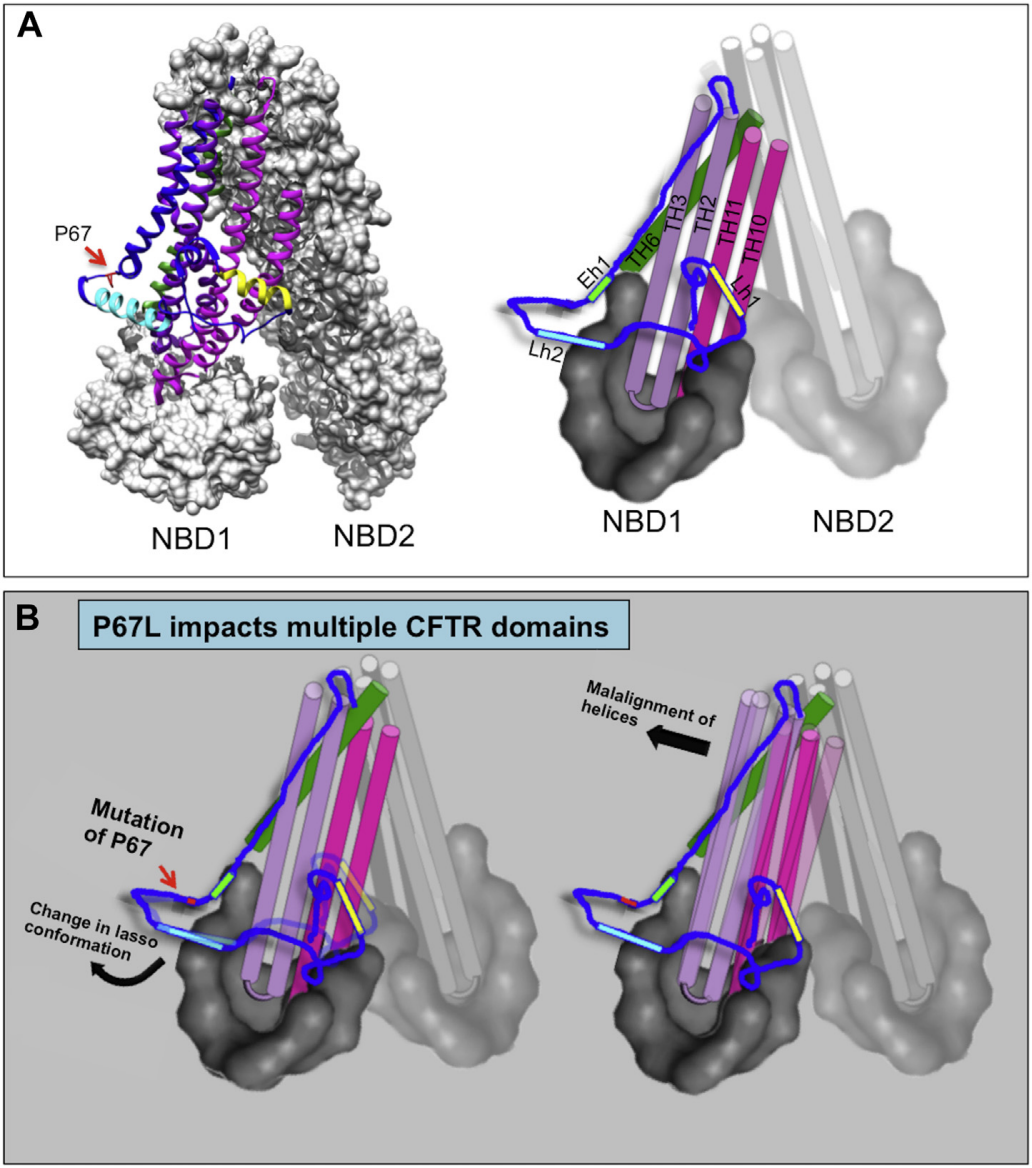
**Model depicting structural defects conferred by P67L.***A*, the *ribbon* diagram on *left* demonstrates lasso arrangement relative to the transmembrane domains (TMDs). Lasso helix 1 (Lh1), lasso helix 2 (Lh2), and elbow helix 1 (Eh1) are shown (detail) on *right*. The lasso motif serves to constrain adjacent TMD helices (*e.g.*, TMD1–TH2, TH3, and TH6) and (TMD2–TH10 and TH11). In panel (*B*), mutation of P67 leads to changes of an alpha-helical “kink” near the proline, contributing to disruption of lasso position and misalignment of transmembrane helices, global conformational change from loss of TMD structure, and overall protein misfolding/early degradation by the proteasome. Clinically important CFTR mutations near P67L (E56K and W57G) would also be expected to impact folding of the lasso motif. CFTR, cystic fibrosis transmembrane conductance regulator.

The present studies suggest a model in which point mutations in the amino terminus (*e.g.*, P67L) disrupt folding of a large transmembrane protein, in a manner reversed by CFTR pharmacocorrection (Fig. 9). We expect other CFTR class II variants mediate pathogenesis in similar fashion and may exhibit commonalities in the same misfolding program (*i.e.*, sequential domain instability because of key positions in CFTR that govern multidomain folding, including involvement of TMD1 and Lh2 during lumacaftor rescue). Our data support a mechanism in which specific transmembrane alpha helices encoded by TMDs 1 and 2 are maintained in proper orientation by Lh2, and that loss of this constraint leads to misfolding of downstream CFTR elements. In that context, further studies will be necessary to determine precise ways in which Lh2 interacts with transmembrane alpha helices and thereby maintains proper conformational stability of CFTR.

## Experimental procedures

### Sequence alignment generation

Conservation of N-terminal CFTR residues (aa 1–109, containing Lh1, Lh2, El1, and TH1 based on published human CFTR structure (12)) across 11 species (UniProt KB IDs: UniProt no. P13569-1, UniProt no. Q2IBF9, UniProt no. A0A0G2STD8, UniProt no. Q1LX78, UniProt no. P35071, UniProt no. Q6PQZ2, UniProt no. Q07E16, UniProt no. Q2QLA3, UniProt no. Q2QLA3, UniProt no. Q00554, and UniProt no. P26363) was evaluated. Sequence homology was calculated through a ConSurf analysis derived from the UniRef90 database (http://consurf.tau.ac.il/2016/) (72). HMMER (http://hmmer.org) was used to identify homologous protein or nucleotide sequences and to perform sequence alignments. Based on an HMMER E-value of 0.0001 and 3 iterations, the maximal and minimal percent ID between sequences was 95 and 35, respectively. Two hundred six sequences were sampled from the list of homologs to the query yielding 150 unique HMMER hits. Conservation score was calculated by a Bayesian method and the model of substitution for proteins based on best fit. Similarity of amino acid sequences *versus* the human CFTR sequence query was computed using the R package “stringdist” and scored according to Damerau–Levenshtein distance. Using this metric, sequences that require fewer amino acid transpositions to equal the query sequence were scored higher. Representative species indicating a range of conservation and genera were selected to highlight CFTR N-terminal homology.

### FRT cell line generation and transient transfection

FRT parental cells were obtained from Dr M. Welsh (University of Iowa, Iowa City) and modified using the Flp-in system (Thermo Fisher) to express CFTR. FRT flp host clonal cells were established by transfecting pFRT/lacZeo and selecting Zeocin-resistant cells. A series of *CFTR* complementary DNAs were cloned into NheI and XhoI sites of the pcDNA5/FRT expression plasmid as described previously (7, 17) and cotransfected with plasmids containing Flp recombinase (pOG44) to obtain target site-specific genomic insertion. Similar levels of CFTR mRNA transgene were confirmed by droplet digital PCR for cell lines studied here (7, 17). For transient expression of CFTR in the FRT model, FRT parental cells were seeded at 150,000 cells per 0.33 cm^2^ transwell filter 2 days prior to transfection. Plasmid DNA (0.5 μg) was suspended in 0.5 μl of Lipofectamine 3000 (Thermo Fisher Scientific) together with 0.75 μl P3000 reagent in 20 μl Opti-MEM (Gibco) for 20 min and applied to each filter for 5 min 1 day prior to short-circuit current analysis.

### Cell culture

FRT cells were maintained in Coon's Modified Ham's F12 (Sigma–Aldrich), supplemented with 5% fetal bovine serum (FBS) and grown at 37 °C in a humidified incubator containing 5% CO_2_/95% O_2_. For rescue and delivery of F508del and P67L CFTR to the plasma membrane, cells were incubated with 3 μM lumacaftor (Selleck Chemicals) at 27 °C for 24 to 48 h.

HEK293 cells for Western blot analysis were cultured and transfected as previously described (7). For limited proteolysis studies, HEK293 cells were maintained in Dulbecco's modified Eagle's medium supplemented with 10% FBS (qualified, European Union–approved, South America origin) and 2 mM Glutamax at 37 °C with 5% CO_2_/95% O_2_. HEK293 cells were grown in 6-cm dishes to ∼70% confluency, and plasmid DNA was transfected using PEI (1 mg/ml). Five micrograms of DNA was added to 200 μl of 150 mM NaCl and 12.5 μl PEI and incubated for 10 min at room temperature. Cells were then grown in Dulbecco's modified Eagle's medium supplemented with 5% FBS, and 1 mM Glutamax, to which DNA:PEI complexes were added. After 4 h, supernatant was aspirated and replaced with full-growth medium.

### Antibodies and chemicals

Anti-CFTR mouse monoclonal antibodies used here were 10B6.2 antihuman NBD1 (http://www.cftrfolding.org/reagentRequests10B62.asp), UNC570R domain (directed against CFTR aa 731–742), 596 NBD2 (aa 1204–1211), 769 NBD2 (aa 1204–1211), 432R domain (aa 762–770) (http://www.unc.edu/∼tjjensen/CFADP/) (from J. Riordan, University of North Carolina, Chapel Hill), and 24-1 anti–C-terminal monoclonal (R&D Systems). Rabbit polyclonal Mr. Pink was raised against human NBD1 (30, 31, 32), and antisera TMD1C and TMD2C were generated in rabbits against keyhole limpet hemocyanin–coupled peptides of the C termini of TMD1 and TMD2, respectively (31, 32). Polyclonal anti–β-actin antibody (Sigma–Aldrich; catalog A5441), horseradish peroxidase–conjugated goat antimouse IgG, and anti-rabbit IgG (Bio-Rad [catalog numbers: 1706516 and 1721034, respectively]) were utilized for immunoblotting. SuperSignal West Pico chemiluminescence substrate was from Thermo Scientific and CFTR potentiator (ivacaftor) from Selleck Chemicals. Chemicals were purchased from Sigma–Aldrich, Thermo Scientific, or as described later.

### Western blot analysis

Cells were washed with PBS, pelleted, and lysed with radioimmunoprecipitation buffer (150 mM NaCl, 0.1% Triton X-100, 0.5% sodium deoxycholate, 0.1% SDS, 50 mM Tris–HCl, pH 8.0) containing protease inhibitor cocktail and EDTA for 45 min on ice. Protein concentration was evaluated using a Bicinchoninic acid assay (Thermo Scientific). For these studies, 40 μg cell lysate from each sample was resolved by SDS-PAGE and transferred to polyvinylidene difluoride membranes, followed by incubation with specific primary antibody and horseradish peroxidase–labeled secondary antibody. Chemiluminescent substrate (SuperSignal West Pico; Thermo Scientific) was used for development and signal quantified by a ChemiDoc XRS (Bio-Rad), with densitometry performed using ImageJ software (National Institutes of Health: https://imagej.nih.gov) or ImageQuant (Bio-Rad). Based on numerous past studies of gel migration, the fully glycosylated CFTR represents a predominantly post-ER form, whereas a core CFTR glycoform is taken as localized to the ER or early trans-Golgi network (3, 10, 11, 25, 73).

Quantitation of CFTR protein transiently expressed in 293 cells was normalized to CFTR mRNA expression level in simultaneously transfected duplicate samples. mRNA levels were measured by droplet digital PCR (Bio-Rad) using *CFTR* (6-carboxyfluorescein–labeled target) and hypoxanthine phosphoribosyltransferase 1 (hexachloro-fluorescein-labeled reference) specific probes, and following manufacturer's suggested protocol.

### Electrophysiology

Voltage clamp was measured with an MC8 instrument and P2300 Ussing chamber set up (Physiologic Instruments) configured for short-circuit current (I_sc_) monitoring. Cell monolayers expressing CFTR were incubated bilaterally with Ringer's solutions containing the following (in millimolar): 115 NaCl, 25 NaHCO_3_, 2.4 KH_2_PO_4_, 1.24 K_2_HPO_4_, 1.2 CaCl_2_, 1.2 MgCl_2_, and 10 d-glucose (pH 7.4). Three-millivolt pulses were applied every 10 s to calculate resistance by Ohm's law. In some experiments, mucosal buffer was switched to low Cl^−^ solution (1.2 mM NaCl and 115 mM Na^+^ gluconate, with other components as mentioned previously) to increase electrochemical driving force for chloride secretion. Amiloride (100 μM) was used to inhibit residual Na^+^ currents, and the agonist forskolin (5–20 μM) and potentiator ivacaftor (5 μM) were added to stimulate ion transport through CFTR. Inh_172_ (10 μM), a CFTR inhibitor, was used in the apical solution at the completion of experiments to block CFTR-dependent I_sc_. All chambers were maintained at 37 °C with perfusion of 5% CO_2_/95% O_2_ while measurements were made.

### Pulse-chase analysis

Twenty-four hours after transfection, HEK293T cells were studied by pulse chase as described previously (30, 31, 74). Briefly, cells were labeled for 10 min with 143 μCi/6-cm dish of EasyTag Express ^35^S protein-labeling mix (PerkinElmer). Incorporation of the label was stopped by adding a 5 mM excess each of unlabeled cysteine and methionine. At indicated chase times, dishes were transferred to ice and washed twice with ice-cold Hanks' balanced salt solution (Life Technologies). Cells were lysed with 1% Triton X-100 in 20 mM MES, 100 mM NaCl, 30 mM Tris–HCl, pH 7.5. Lysates were cleared of nuclei by centrifugation for 10 min at 16,000*g* at 4 °C. Then, the supernatant was divided for either limited proteolysis or direct immunoprecipitation of the full-length protein as described (30, 31, 75). Where indicated, VX-809 (lumacaftor) was added to a 3 μM concentration in culture media during starvation, pulse, and chase.

### Limited proteolysis and immunoprecipitation

Limited proteolysis was conducted as described (30, 31, 33). In short, detergent cell lysates from the pulse chase were digested for 15 min on ice, using 25 μg/ml proteinase K (Sigma). Digestion was stopped by addition of an equal volume of 20 mM MES, 100 mM NaCl, 30 mM Tris–HCl, pH 7.5 with 1% Triton X-100 containing 2 mM PMSF and 2 μg/ml of chymostatin, leupeptin, antipain, and pepstatin A (Sigma).

Following proteolysis, equal aliquots of the digested lysates were transferred to 50 μl of Protein-A Sepharose beads (GE Healthcare), which had been preincubated with antibody for 10 min at 4 °C. All immunoprecipitates were washed twice for at least 10 min at room temperature. Eight microliters of TMD1C antibody, added to Protein-A beads, was used to isolate all fragments originating from the TMD1 domain. Lysates with the antibody and beads were incubated for 3 h at 4 °C and washed twice with 10 mM Tris–HCl pH 8.6, 300 mM NaCl, 0.05% SDS, and 0.05% Triton X-100. NBD1 fragments were immunoprecipitated with 7.5 μl of Mr. Pink polyclonal antibody (anti CFTR-NBD1; (30, 31, 32)) and incubated overnight at 4 °C. The immunoprecipitates were then washed twice with 10 mM Tris–HCl pH 8.6, 300 mM NaCl, 0.1% SDS, and 0.05% Triton X-100. TMD2 fragments were immunoprecipitated with 8 μl of TMD2C antibody, incubated for 3 h at 4 °C, and washed twice with 50 mM Tris–HCl pH 8.0, 150 mM NaCl, and 1 mM EDTA. NBD2 fragments were immunoprecipitated with 0.25 μl 596 antibody and incubated for 3 h at 4 °C, followed by two washes with 30 mM Tris–HCl pH 7.5, 20 mM MES, 100 mM NaCl, and 0.5% Triton X-100. Washed immunoprecipitates were resuspended in 10 μl of 10 mM Tris–HCl pH 6.8, followed by 10 μl 2× Laemmli sample buffer containing 40 mM DTT and were heated for 5 min at 55 °C. Samples for full-length protein and proteolyzed fragments were separated by 7.5% or 12% SDS-PAGE, respectively, then dried, and exposed to radiosensitive screens for phosphoimaging.

### Homology modeling

New models of WT-CFTR in the inward- and outward-facing conformations were built based on the available cryo-EM structures. The inward-facing orientation was generated after augmenting the human EM-structure by ∼50 residues based on the zebrafish CFTR using Modeller ((76); Fig. S5). High structural similarity between human (Research Collaboratory for Structural Bioinformatics Protein Data Bank [PDB]: 5UAK) and zebrafish (PDB: 5UAR) inward-facing CFTR provides support for this approach; indeed, the root-mean-square deviation between the human and zebrafish structures is only 2.3 A. The outward-directed model was homology constructed based on zebrafish (PDB: 5W81; 55% sequence identity to human CFTR), again using Modeller. P67L-CFTR and P67L/R555K-CFTR were built using the Mutate Residue plugin available through Visual Molecular Dynamics (University of Illinois at Urbana-Champaign). Resulting structures were subjected to a minimization of 200,000 steps.

### MD simulations

MD analysis was performed using WT, P67L, or P67L/R555K-CFTR initiated both from the inward- and outward-facing conformations. Prior to simulation, structures were processed using protein preparation wizard as implemented in Schrodinger's Maestro, with protonation states of titratable residues based on predicted pKa values.

All structures were simulated within 160 Å × 160 Å or 140 Å × 140 Å membrane patches composed of 1-palmitoyl-2-oleoyl-sn-glycero-3-phosphocholine lipid for inward- and outward-facing conformations, respectively. The initial positioning of helices within the membrane was determined using the Orientations of Proteins in Membranes server (77). All systems were solvated by 3-site transferable potential water (78), with sodium and chloride ions added in the aqueous phase to neutralize the system and obtain salt concentrations of 0.15 M using Ionize plugin function in Visual Molecular Dynamics.

MD simulations were conducted with NAMD 2.12 (79) using the CHARMM36 force field (80), including φ/ψ cross-term map correction under Langevin temperature and pressure control, and periodic boundary conditions with particle-mesh Ewald (81) electrostatics. Cutoffs for van der Waals and Coulomb interactions were set to 10 Å. Simulated systems were energy minimized to remove van der Waals clashes. Following minimization, systems were equilibrated over three cycles as follows: (1) lipid membrane tails were allowed to melt for 10 ns, whereas all other components of the systems were fixed in order to introduce the suitable disorder of a fluid-like bilayer; (2) systems were allowed to equilibrate for 20 ns while constraining the positions of the protein atoms; and (3) systems were allowed to equilibrate for 20 ns with no constraints. Following equilibration, production phases were carried out at a constant temperature of 310 K, constant pressure of 1 atm, and integration time step of 2 fs. Each structure was simulated three times for 100 ns, each time starting from a different random seed. Simulations were analyzed primarily by examination of the resulting root-mean-square deviation and root-mean-square fluctuation plots.

### Statistics

Data were summarized as mean ± SEM. Two-sample *t* tests were used to assess statistical significance of observed differences in mean response across two conditions applied to a particular assay. *p* Values of less than 0.05 were considered significant.

## Data availability

All data are contained within the article and/or as supporting information.

## Supporting information

This article contains supporting information (Supplemental Data 1 and 2) (12, 43).

## Conflict of interest

The authors declare that they have no conflicts of interest with the contents of this article.
