# Supplementary material for: The CFTR P67L variant reveals a key role for N-terminal lasso helices in channel folding, maturation, and pharmacologic rescue
Source: J Biol Chem. 2021 Mar 26;296:100598. doi: 10.1016/j.jbc.2021.100598 (PMC8102917; doi:10.1016/j.jbc.2021.100598)
Supplement: Supplemental Figures S1–S5 [file mmc1.docx]

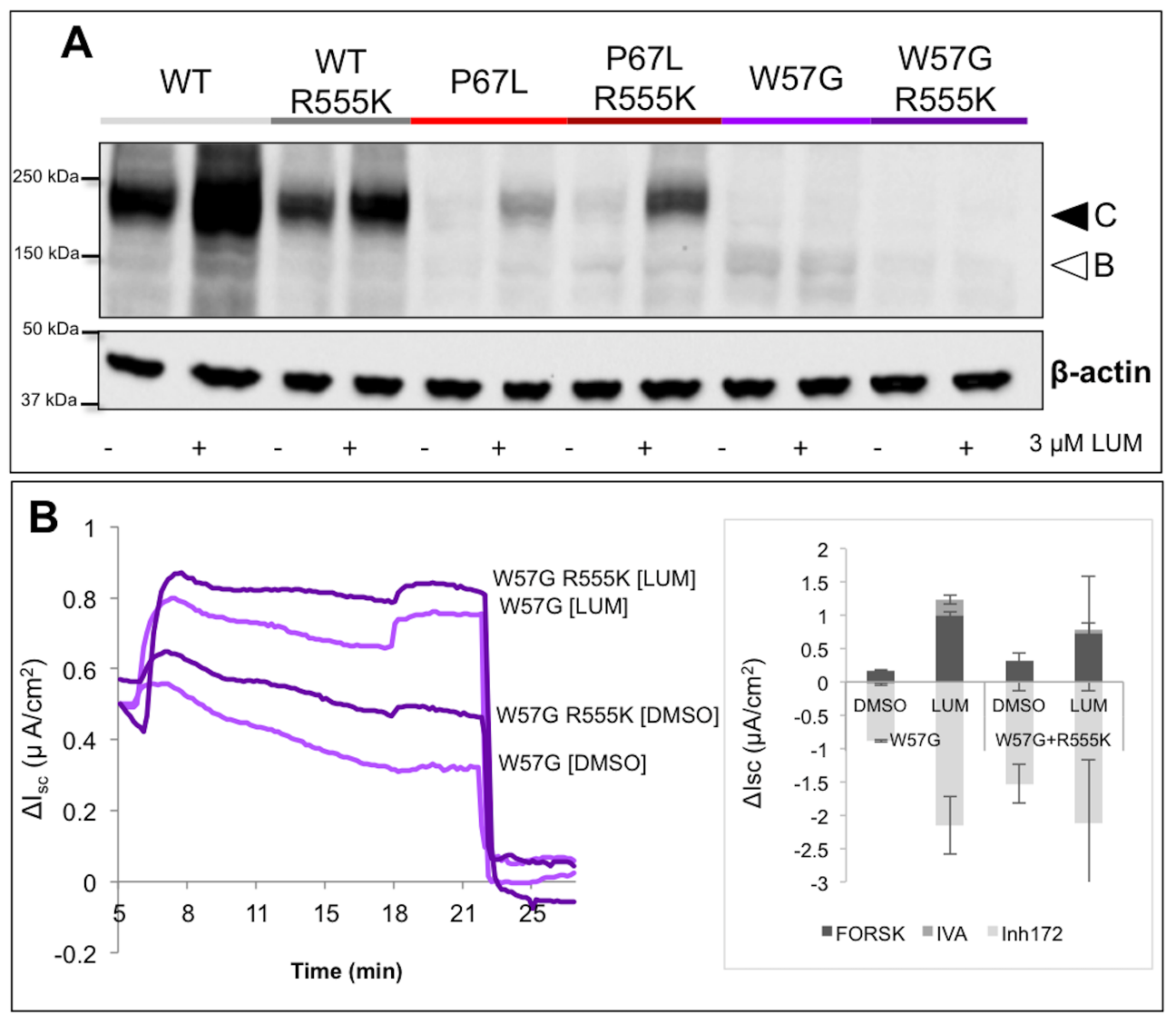


**Figure S1. W57G, an Lh2 point mutation, is completely resistant to lumacaftor and second-site suppression**. (A) In contrast to P67L (and E56K, Fig. 3), W57G, expressed transiently in HEK293 cells, demonstrates no response to lumacaftor and/or R555K correction. Lumacaftor treatment (VX809, 3 µM) was for 24 hours; CFTR detection was with UNC596 and UNC570 antibodies. (B) Ussing chamber studies applied to W57G CFTR in FRT cells. Note change in y-axis compared with Figure 2. Short circuit current for this variant is barely detectable by the Ussing chamber protocol, consistent with absence of CFTR protein and/or negligible inh172-inhibited constitutive function. The experiment has been repeated twice with similar results. The W57G mutation is a clinically significant abnormality found in a small number of individuals with CF (<https://cftr2.org/>). Error bars show standard deviation.

**
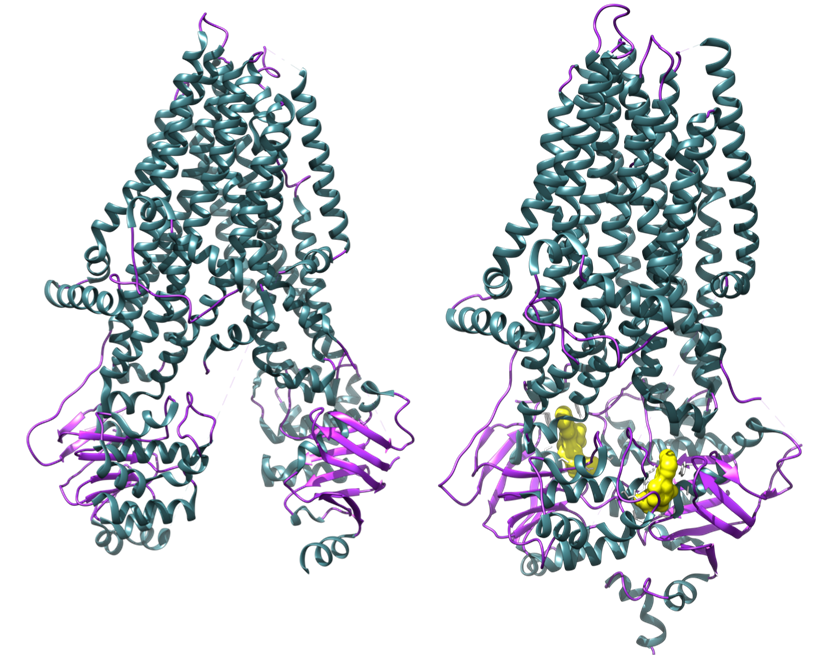
**

**Figure S2. Models of WT-CFTR used for MD simulations** based on the (A) non-phosphorylated (without ATP), inward facing [PDB # 5UAK](12) and (B) phosphorylated, ATP-bound (shown in yellow), outward facing [PDB # 5W81](43) cryo-EM structures. Purple indicates unstructured regions and beta-sheets while dark blue represents alpha helices.

#
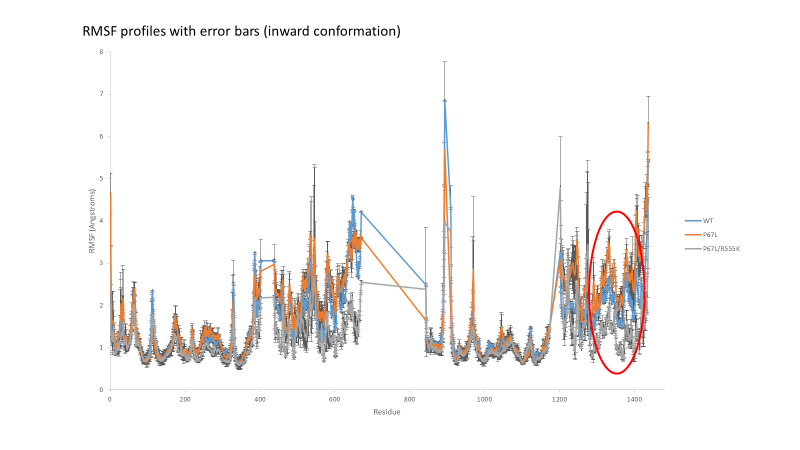


**Figure S3. RMSF profiles with error bars (inward conformation).** RMSF profiles averaged (over three 100 ns simulations) for WT (blue), P67L (orange) and P67L/R555K (grey) CFTR are shown. Error bars for each data point are provided, which represent the standard deviation of RMSF values over three simulations of 100 ns.


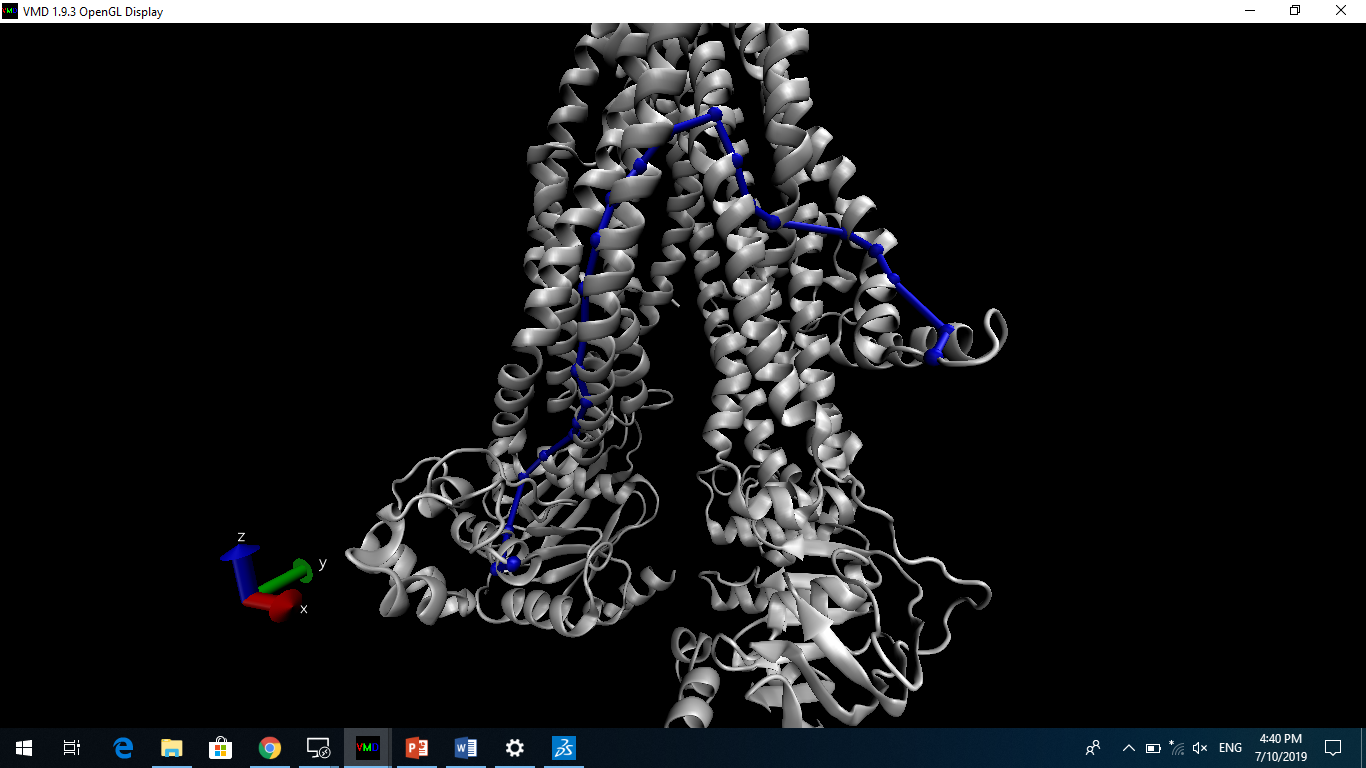

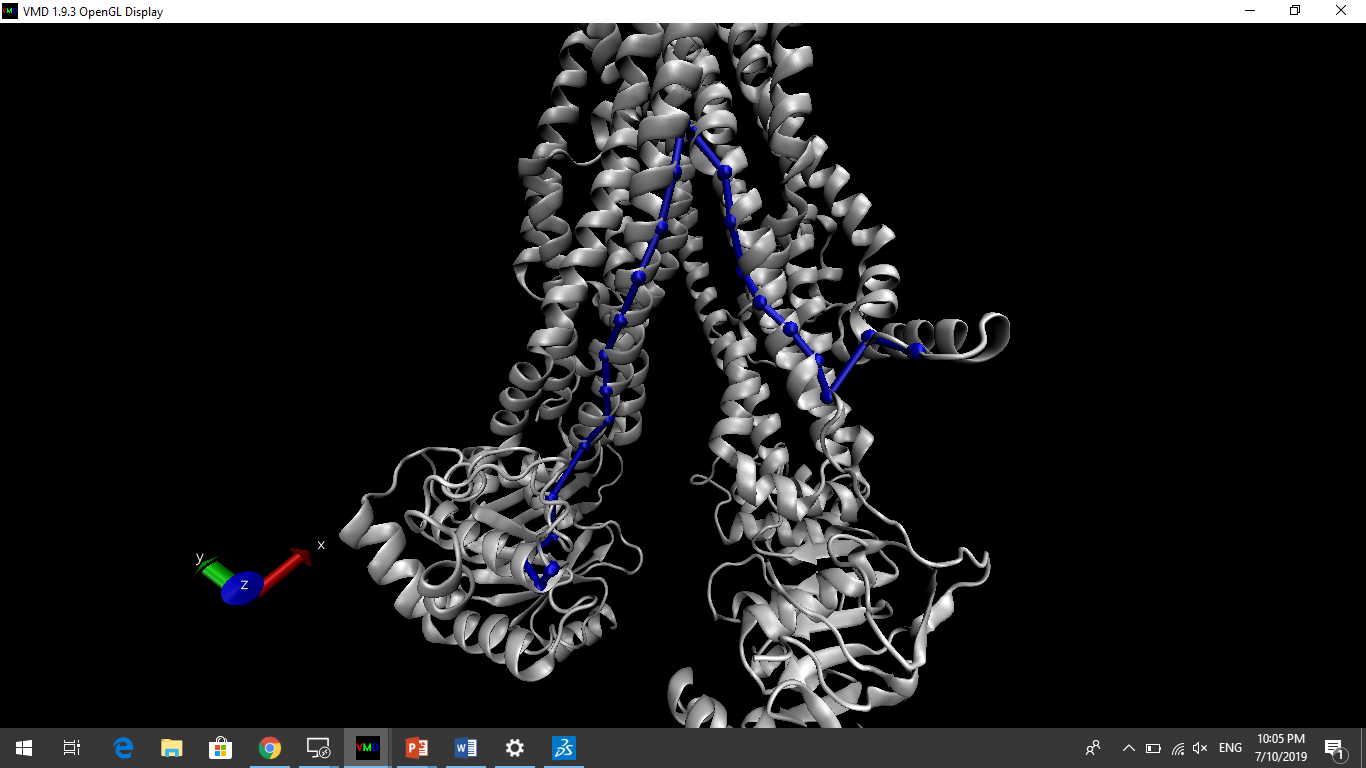


**Figure S4. Dynamic network analysis for P67L and WT CFTR**. Dynamic network analysis was applied to map allosteric pathways for WT (left) and P67L (right) CFTR. In the inward facing conformation, where destabilization of NBD2 is more evident, a path between residues at positions 67 and 1347 (the position for which the largest RMSF difference between the WT and the mutant proteins was observed) was identified. This path involves residues L67, K68, L375, Q372, N369, G366, D363, Q359, P355, T351, A234, A238, R242, K246, Q250, K254, E257, V260, E264, F1294, P1290, Q1352, G1349, and S1347. A similar path between P67 and S1347 was observed for the WT protein involving residues P67, N66, R75, F78, F81, A198, A357, F354, V350, A234, Q237, L240, M243, R251, K254, E257, I261, M265, V272, Y275, C1355, 1350, and S1347. In WT CFTR, residues Y275 and C1355 mediate a stabilizing H bond interaction between MSD1 (Y275) and NBD2 (C1355), which is maintained throughout ~54% of the simulation. This interaction is missing in P67L-CFTR, where residues Y275 and C1355 are not part of the pathway, possibly accounting for the destabilization of the NBD2 in the mutant. Interestingly, residues E264 and F1294, which connect MSD1 and NBD2 along the mutant pathway, are not involved in any stabilizing interaction.


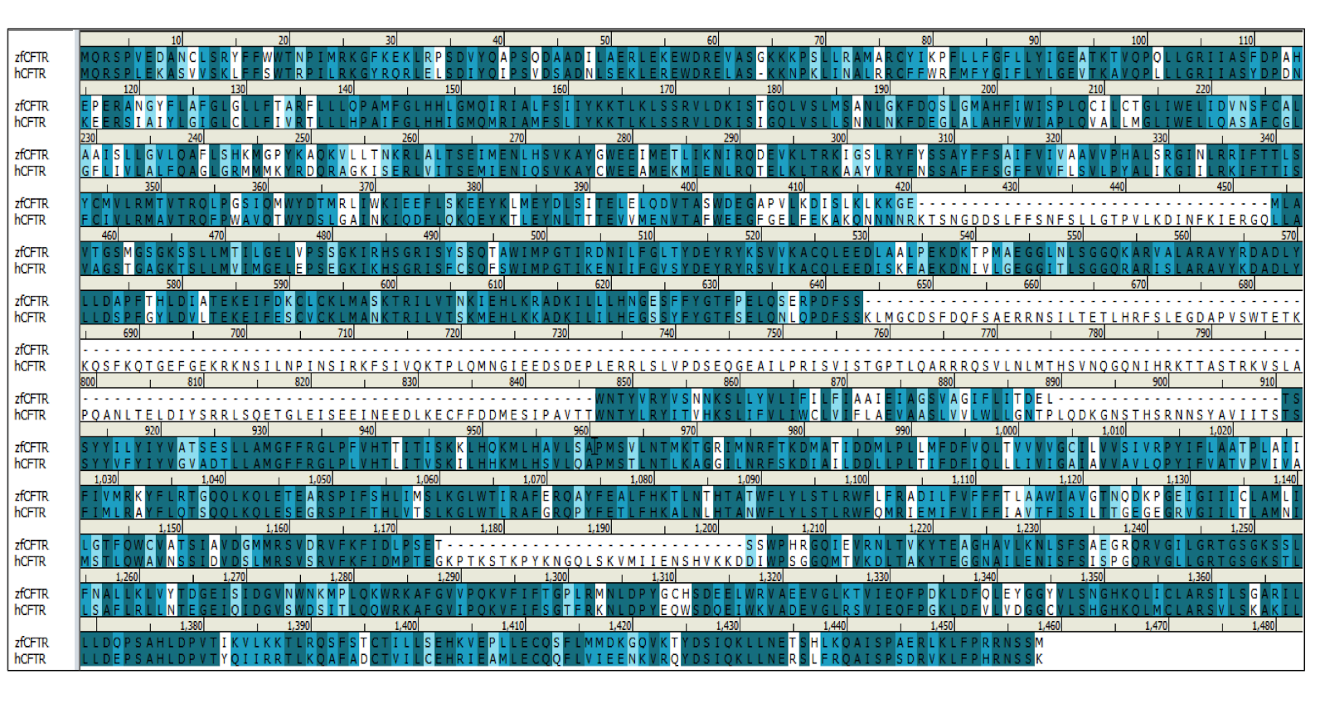


**Figure S5. Sequence alignment between zebrafish and human CFTR.**  Identical, similar and non-similar residues are depicted in dark blue, light blue, and white, respectively. Coordinate files are provided as a Supporting Information data attachment.
